# Clinical, laboratory and neuroimaging profile of patient's cohort with septo-optic dysplasia treated at a pediatric university hospital

**DOI:** 10.1016/j.jped.2024.08.009

**Published:** 2024-10-10

**Authors:** Tabatha P.C. Braga, Izabel C.R. Beserra

**Affiliations:** Faculdade de Medicina da Universidade Federal do Rio de Janeiro (UFRJ), Programa de Pós-Graduação (PPG) em Medicina (Endocrinologia), Rio de Janeiro, RJ, Brazil

**Keywords:** Septo-optic dysplasia, Hypopituitarism, Endocrine system, Optic nerve hypoplasia, Child

## Abstract

**Objectives:**

Septo-optic dysplasia (SOD) is a relatively rare clinical condition. However, there has been a significant increase in its incidence over the years. Diagnosis is clinical and made when there are at least 2 components of the classic triad: Optic nerve hypoplasia (ONH), midline malformation, and pituitary dysfunction. This study aims to describe the clinical and complementary exam characteristics of patients with SOD.

**Methods:**

A retrospective study of review of medical records of 48 patients cohort (24 female) with SOD followed to 2023.

**Results:**

The average age at diagnosis was 3.90 ± 3.85 years. Maternal age was ≤ 25 years at the time of delivery in 50% (24/48) of cases. Visual and developmental impairment was observed in 21 (43.7%) and nystagmus in 15 patients. Fourteen of them developed short stature. Regarding the diagnostic criteria for SOD: 92.6% (38/41) had ONH (78.9% bilaterally), 95.3% (41/43) had structural midline abnormalities, 85.7% (24/28) had hypothalamic-pituitary region alterations, and 73% had at least one hormonal deficiency, of which 2/3 had multiple pituitary dysfunctions. The most frequent deficiencies were thyroid-stimulating hormone and growth hormone, and the average age at diagnosis of the first dysfunction was 4.25 ± 3.71 years.

**Conclusion:**

Clinical manifestations that most led to early suspicion were developmental delay, nystagmus and visual impairment. More than 1/3 of the patients had the complete triad and 2/3 developed multiple pituitary deficiencies, with TSH deficiency being the most prevalent followed by GH deficiency. Patients with ONH or midline structural changes should undergo endocrine evaluation.

## Introduction

Septo-optic dysplasia (SOD), also known as Morsier's syndrome,^1^ is a relatively rare clinical condition, with a prevalence of 1:10,000 to 20,000 live births.^2^ However, there has been a significant increase in its incidence over the years,^3^^,^^4^ making it imperative to widely disseminate knowledge about this condition among the medical community.

Its diagnosis is clinical and established when at least two components of the classic triad are present: optic nerve hypoplasia (ONH), midline malformation, and pituitary dysfunctions.^5^ ONH identification occurs through fundoscopy, where a small and pale optic disc is observed.^6^ Midline malformation can be detected through neuroimaging exams such as magnetic resonance imaging (MRI) and cranial tomography (CT), with alterations in the corpus callosum and septum pellucidum being the most commonly found.^5^ Finally, the diagnosis of pituitary dysfunctions is confirmed through laboratory tests measuring hormones involved in the hypothalamus-pituitary axis.^7^

Hormonal dysfunctions are present in the majority of patients with SOD, affecting up to 70%. Growth hormone (GH) deficiency is the most prevalent among the hormonal dysfunctions found in SOD^8^ Therefore, it is mandatory for these patients to be under the care of an endocrinologist for evaluation, follow-up, and treatment of possible pituitary disorders.

SOD is a rare clinical condition and often undiagnosed. Knowledge of its clinical, laboratory, and imaging characteristics is crucial for its early identification, enabling the diagnosis of pituitary dysfunctions and an immediate establishment of necessary treatment and appropriate follow-up.

The present study aims to describe the clinical and complementary exam characteristics of the patient's cohort diagnosed with SOD who are under follow-up in a pediatric endocrinology outpatient clinic.

## Material and methods

This is a retrospective longitudinal study of a review of medical records of patients diagnosed with SOD, treated at the endocrinology outpatient clinic of a pediatric university hospital from 2010 to September 2023.

The following data were collected: gestational age (GA) and classification for GA, maternal age at birth, signs and symptoms presented in the first consultation, age at the time of referral to the pediatric endocrinology outpatient clinic, and age at diagnosis of SOD, presence of unilateral or bilateral optic nerve hypoplasia, structural alterations and hypothalamic-pituitary region showed in radiological images of the skull/brain, presence of hormonal dysfunctions and age of presentation, anthropometrics measures and age of onset of pubertal development

The diagnosis of SOD is clinical and was established when at least two components of the classic triad (ONH, midline malformation, and pituitary dysfunction) were present. ONH was assessed through direct fundoscopy conducted by an ophthalmologist in which morphologic ophthalmic anomalies were found, such as a small optic disc, pallor of the optic disc, double-ring sign, abnormal vascular pattern, small size of the optic disc, or a small neuroretinal rim area. ONH was confirmed with measurements of the optic disc, where the ratio of the horizontal disc diameter (DD) to the distance between the macular and the temporal edge of the disc (DM) less than 0.35 suggests ONH; midline malformations were diagnosed using neuroimaging exams–CT or MRI or Transfontanelle Ultrasound (TFUS), reviewed by a radiologist for specific malformations such as absence of the septum pellucidum; hypoplasia of the corpus callosum; abnormalities of the pituitary gland (absent infundibulum, ectopic, or absent neurohypophysis or absent adenohypophysis); and other major malformations (hydrocephalus, schizencephaly, holoprosencephaly or white matter hypoplasia); and hormonal dysfunctions were evaluated through evaluation clinical and serum hormone assays carried out periodically during the follow-up in the pediatric endocrinology outpatient clinic.

The categorical variables were described in simple frequency distribution, calculations of mean and standard deviation score (SDS), and minimum and maximum values. The results obtained are presented in tables and graphs.

This study followed the Guidelines and Regulatory Norms for Research Involving Human Subjects, in accordance with CNS Resolution No. 466/2012 and its complementary resolutions, and the Medical Ethics Code, CFM Resolution No. 1931/2009, Articles 99 and 110. There are no conflicts of interest, and this research did not require financial resources for its completion. The study was approved by the Research Ethics Committee of the institution.

## Results

The medical records of 48 patients (24 female and 24 male) diagnosed with SOD were selected, 28 of which had regular follow-up at the outpatient clinic.

The average age at the time of referral to the pediatric endocrinology outpatient clinic was 3.15 ± 3.30 years (0.08–11.16 years), with 39.58% being referred in the first year of life. The mean age at diagnosis of SOD was 3.90 ± 3.85 years (0.08–15 years). Considering the patients still under follow-up (*N* = 28), the current average age is 7.46 ± 4.83 years (0.66–15.5 years).

**Figure 1:**
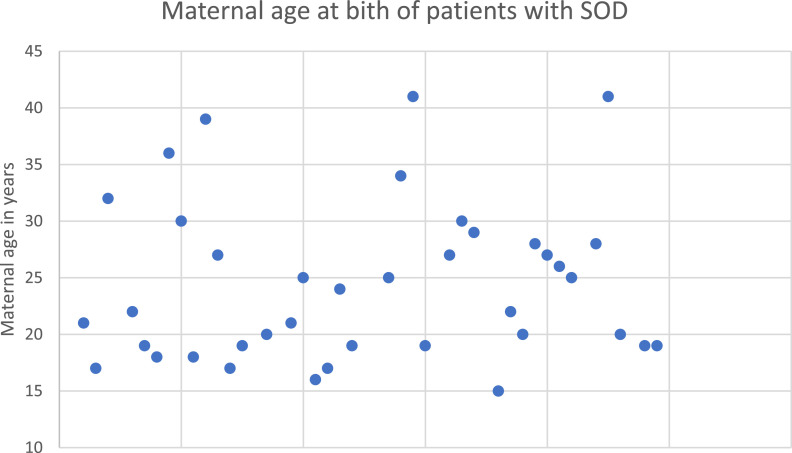
Maternal age at birth of patients with SOD excluding nine patients, from whom it was not possible to obtain such information.

Regarding perinatal data, 44.44% (*n* = 20/45) of the patients were born to primiparous mothers, while 53.33% (*n* = 24) were not. For the remaining 3 patients, this information was not available. Maternal age at birth is depicted in Figure 1. The average gestational age at birth was 37.39 ± 3.58 wk (25–42), with 8 (16.7%) being preterm born.

The signs and symptoms identified in the first consultation at the endocrinology outpatient clinic are presented in Table 1.

**Table 1:** Signs and symptoms (A), Structural changes found in radiological examinations of the skull/brain (B) and of sella turcica (C) in cohort of patients with SOD.

A - Clinical features	Frequency (%)	(*N*)
Visual deficiency	43.7	21
Developmental delay	43.7	21
Nystagmus	31.3	15
Short stature	29.2	14
Cholestasis or jaundice	25.0	12
Strabismus	25.0	12
Convulsion	25.0	12
Hypoglycemia	25.0	12
Micropenis	20.8	5/24
Microcephaly	14.6	7
Hypernatremia	10.4	5
Obesity	10.4	5
Cleft palate	8.3	4
Hearing deficiency	4.2	2
B - Structural changes in skull//brain neuroimaging		
Pellucid septum^a^	53	23
Corpus callosum^a^	49	21
Optic chiasm	23	10
Schizencephaly	11.6	5
Optic nerve	9.3	4
Cortical atrophy/dysplasia	7	3
Cerebellum	7	3
Holoprosencephaly	4.5	2
Lisencephaly	4.5	2
Calcifications	4.5	2
Gliosis	4.5	2
Olfactory bulb	2.3	1
C - Sella neuroimaging		
Hypoplastic anterior pituitary	65.7	16
Ectopic or absent neurohypophysis	58.3	14
Tapered or absent pituitary stalk	45.8	11
Empty sella	4.2	1

a Hypoplastic or absent.

Forty-one patients underwent fundoscopy, of which 92.6% (*N* = 38) had optic nerve hypoplasia: 30 (78.9%) bilaterally and 8 (21.0%) unilaterally. The average age at the diagnosis of optic nerve hypoplasia was 2.33 ± 3.42 years (0.08–15 years).

Radiological images of the skull/brain (CT or MRI or TFUS) were performed on 43 patients, with 95.3% (*N* = 41) showing structural alterations (Table 1).

Hypothalamic-pituitary region radiological images were obtained from 28 patients, among whom 85.7% (*N* = 24) exhibited structural alterations (Table 1).

**Figure 2:**
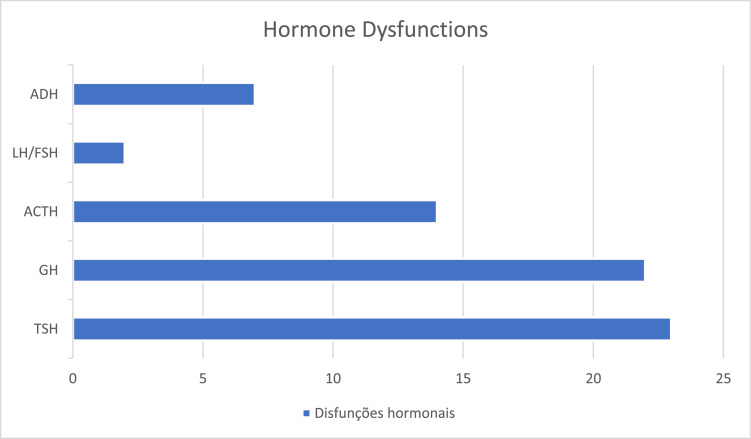
Number of patients with each hormonal dysfunction (TSH, GH, ACTH, Gonadotropins and antidiuretic hormone -ADH).

Regarding hormonal dysfunctions, 73% (*N* = 35) had at least one deficiency, of which 2/3 had multiple pituitary dysfunctions. The most prevalent deficiency was thyroid-stimulating hormone (TSH) deficiency, as shown in Figure 2. The average age at the diagnosis of the first hormonal dysfunction was 4.25 ± 3.71 years (0–12 years).

It was observed that 37.5% (*N* = 18) of the patients met all 3 criteria of the classic triad of SOD.

Considering the patients who are being followed up at the endocrinology outpatient clinic (*N* = 28), the authors observed that 46.4% (*N* = 13) were short stature, 10.7% were overweight and 1 patient (3.5%) was obese. The anthropometric measurements are shown in Table 2.

**Table 2:** Stature, weight and BMI of 28 patients with SOD under followed-up.

Patient	Age (y)	Sex (F/M)	Stature (cm)	Stature SDS^a^	Weight (kg)	Weight SDS^a^	BMI (kg/m²)	BMI SDS^a^
1	4.58	M	92.4	−3.05	13.3	−2.49	15.5	0
2	15.5	M	170.6	−0.18	72.8	1.12	25	1.3
3	13.4	F	156.4	−0.35	40.2	−0.91	16.43	−1.09
4	1.83	F	82.6	−0.86	9.7	−2.3	14.2	
5	4.5	M	114.3	1.97	33.3	3.9	25.48	4.19
6	4.5	M	96.7	−2.1	14.2	−1.74	15.19	−0.29
7	4.33	M	110.4	1.36	19.3	1.01	15.84	0.25
8	1.5	F	78.1	−0.68	9.7	−1.22	15.9	
9	10.08	F	117.9	−3.21	22.3	−2.41	16	−0.42
10	0.88	F	52.5	−0.36	4.3	0.28	15.8	
11	8.32	M	123.73	−1.03	19.9	−2.22	13	−2.65
12	11	M	144.3	0.11	38.8	0.39	18.63	0.58
13	11.75	F	146.6	−0.37	55.8	1.39	25.8	1.78
14	15.16	F	128.2	−5.27	24.8	−7.51	15	−2.6
15	10.88	M	129.7	−1.94	30.3	−0.89	18.01	0.38
16	6.75	M	120.6	0.08	20.9	−0.52	14.36	−0.94
17	1.08	F	70.9	−1.35	6.04	−4.75	12	
18	14.75	F	129.9	−4.93	32.4	−3.5	19.2	−0.2
19	5.75	F	90.1	−5.16	15.2	−2.08	18.7	1.66
20	8.33	F	110.6	−3.41	20.2	−1.84	16.4	0.22
21	12.33	F	128.3	−3.36	27.5	−2.7	16.7	−0.67
22	1.91	F	76.8	−2.35	8.85	−3.08	15	
23	15.16	M	141.2	−3.4	39	−2.41	19.6	−0.13
24	7.5	F	109.5	−2.87	18.6	−1.85	15.3	−0.19
25	4	F	86.45	−3.48	10.6	−3.87	14.18	−1.1
26	0.66	M	66.3	−1.49	7,75	−1.15	17.6	
27	7.3	M	106.4	−3.21	17	−2.82	15	−0.4
28	5.25	F	117.1	1.52	21.4	0.93	15.6	0.32

SDS, standard deviation score.

a SDS for BMI is not classifiable in children under 2 years old by the CDC.

Regarding pubertal development (Tanner stage) of these patients, the mean age at the start of puberty was 7.2 years in girls (ranging from 1.58 to 14 years) and 8.83 years in boys (ranging from 8.16 to 15.5 years). Sixteen patients were still prepubescent, and for 4 patients it was not possible to define the age of onset. Two girls started puberty before the age of 8, and 3 boys initiated puberty before the age of 9.

## Discussion

Forty-eight patients with SOD were identified, demonstrating an increase in casuistic of almost 10 times when compared to a previous study carried out in the same center in 2010.^9^ In studies carried out around the world in recent years, a dramatic increase in the annual incidence of SOD has been observed.^4^^,^^10^ Some authors demonstrate an increase of up to 800% in the last two decades.^4^ However, it is not possible to confirm the existence of a real increase in the incidence of cases, due to the design of this study.

It was possible to identify that there was no difference in frequency between sexes, as found in a European multicenter study^10^ and confirmed in studies around the world, like Webb & Dattani.^5^ Regarding the age of diagnosis of SOD, a mean age of 3.90 ± 3.85 years was found, which is much higher than that found in studies of the Canadian population^4^ (average of 7.5 months) and the European population^10^ (average of 2 months, with 40% diagnosed during prenatal care). This can be explained by a lack of knowledge of the malformation among most general practitioners, who do not suspect it despite the presence of suggestive clinical or imaging findings, leading to a delay in diagnosis. Furthermore, another possible cause for the late suspicion of diagnosis is the lack of appropriate prenatal monitoring, with poor access to exams, such as a quality obstetric ultrasound in order to diagnose midline anomalies such as agenesis or hypoplasia of the corpus callosum or septum pellucidum.^11^

The etiology of SOD is still poorly known, with some genetic anomalies such as HESX1 and SOX2 mutations already identified,^5^^,^^8^^,^^12^ but found in only a small number of cases. It is estimated that known mutations are responsible for only 1% of SOD cases.^13^^,^^14^ Therefore, studies have attempted to evaluate the environmental and biological factors, mainly perinatal, associated with and that may justify the occurrence of SOD.^12^ In this study the authors identified a high prevalence of primiparous women and pregnancies under 25 years of age, similar to what has been observed in research around the world since 1979.^2^^,^^4^^,^^10^^,^^12^ There are few congenital conditions that associate younger maternal age with a higher prevalence, with gastroschisis being the main and best-established one.^10^ However, research has increasingly also shown a strong association between low maternal age with SOD.^10^

Regarding gestational age at birth, there was not a higher prevalence of preterm births, which opposes a Canadian study,^4^ but reinforces the results found by Riedl et al.^15^ Generally, an increased risk of premature birth is observed when there are congenital anomalies present in the fetus.^10^ However, there is no consensus in the literature about this higher prevalence of prematurity in SOD.

The authors identified a high frequency of signs and symptoms in the neonatal period such as hypoglycemia (25%), neonatal jaundice/cholestasis (25%), and micropenis (5 of 14 boys), which could lead to early suspicion of SOD. Neonatal hypoglycemia is an important warning sign when considering hypopituitarism and its early identification with the institution of appropriate therapy is decisive to avoid neurocognitive sequelae.^16^ All newborns presenting such signs should undergo endocrine and ophthalmological evaluation.^17^ In addition to these signs, visual changes such as nystagmus (31.3%), strabismus (25%), and visual deficit (43.7%) were also observed, which strongly lead to the suspicion of ONH and, consequently, of SOD. The development of nystagmus is observed around the 1st to 3rd month of life, followed by strabismus in the 1st year of life.^12^

ONH is one of the components of the classic SOD triad and it is one of the main congenital causes of visual deficits. It is diagnosed by a small and pale optic disc viewed through a fundoscopy examination.^6^^,^^18^ ONH was found in the majority of patients who underwent fundoscopy in our study (38/41 [92.6%]), reinforcing that it is an important risk factor for pituitary endocrine dysfunction and, consequently, for SOD.^18^ Most of the patients had bilateral involvement (78.9%), reinforcing the findings in the literature.^2^^,^^4^^,^^6^^,^^12^^,^^18^ A study demonstrated an average prevalence of 80% of bilateral ONH, and 80% of them presented absolute amaurosis.^12^ Bilateral involvement when compared to unilateral ONH increases the risk of hormonal dysfunction and developmental delay.^5^^,^^12^^,^^18^

Another diagnostic criterion for SOD is the presence of structural changes in the midline, which can be easily identified by computed tomography or magnetic resonance imaging of the skull, or even TFUS if there is an open anterior fontanel. Agenesis/hypoplasia of the septum pellucidum or corpus callosum is the most frequently found, in addition to changes in the optic tract such as ONH or thinning of the optic chiasm.^3^^,^^19^ In the present study, the most frequently found changes were in septum pellucidum (53%), followed by the corpus callosum (49%), optic chiasm (23%), and optic nerve (9%), confirming the literature findings. It is also important to emphasize that neuroimaging exams are not the more adequate methods for diagnosing ONH, with being fundoscopy the specific method.^6^ This justifies a lower percentage of optic nerve changes identified by MRI or CT in other studies when compared to the number of ONH diagnosed in the patients. Other changes identified were schizencephaly (11.6%) and lissencephaly (4.5%), which consist of cortical changes, which when present constitute a condition called SOD plus.^3^^,^^20^ This condition appears to be associated with a developmental delay more severe than that found in SOD without cortical changes.^20^

Regarding the structural assessment of the sella turcica, in most of the patients, the neuroimaging examination showed changes (24/28 [85.7%]). This frequency was higher than described in the literature^21^ but can be justified by the bias that many patients are referred to the outpatient clinic because they already show signs or symptoms of hormonal deficiencies, while those who have not yet manifested such dysfunctions do not arouse the need for referral. Those patients with SOD who present structural changes in the sella turcica already have or are more likely to develop pituitary dysfunction at some point.^21^ However, the finding in neuroimaging of a normal adenohypophysis and neurohypophysis does not exclude the possibility of endocrine changes in the hypothalamic- pituitary axis, with those being present in up to 66% of the patients with normal sella turcica MRI.^21^ Therefore, it is imperative that all patients with SOD, regardless of the results of their neuroimaging exams, maintain follow-up and periodic hormonal evaluation so that possible dysfunctions in the hypothalamic-pituitary axis can be diagnosed.

Regarding pituitary dysfunctions, the third and last diagnostic criterion of SOD, they can manifest themselves as isolated or associated and in varying degrees.^9^ The authors observed that 73% of patients had at least one hormonal deficiency, similar to recent studies with large cohorts.^7^^,^^22^ Researches show that the average age of manifestation of the first pituitary dysfunction in SOD is higher than that in isolated hypopituitarism, which is of 8.54 years.^22^ However, in this study an average age of diagnosis of 4.25 ± 3.71 years was found. This can be explained because the patients referred to the endocrinology outpatient clinic are immediately investigated for changes in the hypothalamic-pituitary axis. Furthermore, another risk factor for the earlier development of deficiencies appears to be the presence of anomalies of the sella turcica (adenohypophysis and/or neurohypophysis), which may also justify the findings of this research, as many of the patients presented such structural changes.

Within pituitary dysfunctions, GH deficiency has been reported to be most prevalent in SOD.^5^^,^^8^^,^^22^^,^^23^ Despite this, the authors observed a higher prevalence of TSH deficiency followed by GH and ACTH, although the difference was not statistically relevant due to the number of patients. However, these findings demonstrate that GH deficiency is not an obligatory component of hypopituitarism and will not necessarily be the first deficiency to manifest itself, reinforcing what the current literature has shown.^22^ The high prevalence of TSH deficiency highlights the importance of its early diagnosis and treatment, since hypothyroidism has been shown to be a significant cause of intellectual deficit, in these children.^18^ Furthermore, central congenital hypothyroidism is commonly not diagnosed in the neonatal screening test,^21^ which reinforces the extreme need to pay attention to this possible deficiency. Any child presenting clinical manifestations suggestive of hypopituitarism, ONH, or midline malformations should undergo endocrine evaluation.^4^^,^^21^

When analyzing the age at diagnosis of each pituitary dysfunction, the authors observed that, despite the low prevalence in the present study and in the literature,^21^ ADH deficiency, leading to diabetes insipidus, was the one with the earliest diagnosis, followed by TSH deficiency. This differs from other research, in which generally the first dysfunction to manifest is the GH deficiency.^8^^,^^22^ This can be explained because to confirm the diagnosis of GH deficiency, in most cases, it is necessary to perform GH stimulation, which can delay the diagnosis. Furthermore, growth deficit will often become more evident only after 3 years of age. Meanwhile, the clinical manifestations of diabetes insipidus are usually very obvious and can lead to hypernatremic dehydration.

Short stature, defined as a height of more than two standard deviations below the average for age and sex, is one of the main reasons for referral to a pediatric endocrinologist.^24^ In this study, 13/28 of the patients had short stature in the follow-up and 1/3 had it since the first medical appointment, which demonstrates its relevance in the diagnosis of SOD. Short stature reflects a growth deficit that can be the first, and often the only, clinical sign of GH deficiency,^24^ which is the main hormonal disfunction in SOD.^5^^,^^8^^,^^22^^,^^23^ In addition, short stature is also a possible clinical manifestation of hypothyroidism,^24^ which is significantly present in SOD, as the authors could see in this study.

As for the BMI classification, the authors find everything from extreme thinness to obesity. Overweight was the initial clinical manifestation in 10.4% (*N* = 5) and only one patient developed obesity during outpatient follow-up. This reinforces that there is no characteristic BMI profile in SOD, unlike what occurs in isolated hypopituitarism, in which there is a greater tendency towards obesity.^22^ However, this contradicts previous studies, which demonstrate a higher prevalence of obesity in SOD, justified by hypothalamic defects that can lead to metabolic changes (hyperphagia, insensitivity to leptin) and favor weight gain.^5^^,^^21^ Both children with weight deficit and those with excess weight require nutritional monitoring.^21^

Finally, patients with SOD are at increased risk of presenting pubertal disorders.^21^ There is a high prevalence of pubertal delay, which, however, is lower than that found in cases of isolated hypopituitarism.^7^ Furthermore, an interesting fact is that precocious puberty (PP) is also observed in patients with SOD.^21^^,^^25^ Its mechanism is not yet fully understood, however, it is suggested that changes in the central nervous system contribute to the disinhibition of the axis, generating excitatory hormone pulses gonadotropin releaser (GnRH).^21^ In the present study, some patients with early puberty were identified.

The clinical manifestations that most frequently led to early suspicion of SOD were developmental delay, nystagmus, visual impairment and, later, short stature. More than 1/3 of the patients presented with the three diagnostic criteria of SOD and 2/3 developed multiple pituitary deficiencies, with TSH deficiency being the most prevalent followed by GH deficiency. Patients with ONH or midline structural changes should undergo endocrine evaluation.

## Financial disclosure

None reported.

## Conflicts of interest

The authors declare no conflicts of interest.
